# Characterisation of a stably integrated expression system for exogenous protein expression in DT40

**DOI:** 10.12688/wellcomeopenres.11816.2

**Published:** 2017-12-12

**Authors:** Meliti Skouteri, Helfrid Hochegger, Antony M. Carr

**Affiliations:** 1 Genome Damage and Stability Centre, School of Life Sciences, University of Sussex, Brighton, BN1 9RQ, UK

**Keywords:** DT40, transcription, promoter, protein expression

## Abstract

The use of constitutive promoters to drive exogenous protein expression is an important tool for the study of diverse biological processes. To create and characterise a stably integrated expression system for DT40 cells, we constructed integration cassettes for three commonly used promoter elements; CMV (cytomegalovirus), CBA (chicken beta actin) or CAG (a hybrid promoter containing the CMV enhancer and chicken beta actin promoter), and used these to stably integrate a *TOPBP1 *transgene at the *OVA* locus, a transcriptionally silent locus commonly used in DT40. We next performed a comparative analysis of protein expression levels and identified CAG as the most efficient of the promoter elements we have tested in DT40 cells. To assess whether the site of integration affected the levels of transgene expression, a second chromosomal locus, immediately adjacent to the endogenous *TOPBP1* gene, was tested for CAG. No major differences in TopBP1 overexpression were observed. This confirms that use of the *OVA* locus for integrating transgenes is a rational choice for DT40. Finally, we demonstrate that our stably integrated overexpression system (SIOS) constructs can be efficiently excised by the induction of tamoxifen-regulated Cre expression. Taken together, SIOS is an easy-to-use and versatile system for constitutive, reversible exogenous protein production that provides a range of potential expression levels. This will be a useful tool for future DT40 experiments.

## Introduction

Exogenously expressing a protein of interest has proved to be a valuable tool during the study of biological processes. A limiting factor for the development of such artificial genetic systems has been the availability of suitably characterised promoter elements. Many constitutive promoters are used during the study of either loss of function (i.e. shRNA) or gain of function (i.e. cDNA expression) systems, as well as systems designed to replace a gene of interest with a mutated copy upon deletion of the endogenous copies, or to produce recombinant protein. However, there are few examples of systematic comparative studies of the expression levels of commonly used promoter elements in specific cell types. Because different experiments have distinct requirements for the level of transgene expression, and because there is limited information available on the efficiency of different promoter systems, the choice of appropriate promoter is often ambiguous: choices are often based on technical convenience or the assumption that, since a particular promoter worked in one cell line, it will work similarly in another.

A characterization of the strengths of six commonly used and randomly integrated constitutive mammalian promoters was reported by Qin *et al.* (2010) in a panel of eight mammalian cell types ^1^. They observed that expression from the cytomegalovirus CMV promoter was the most variable, being strong in some cell types but approximately 7–8 fold lower in others. This finding is consistent with the observation from many other groups that the CMV promoter can become silenced in some cells ^2, 3^. A second important finding by Qin *et al.* (2010) was that the EF1A and CAG promoters were similarly highly expressed, but were more reliable across all the cell lines tested, varying by less than a factor of two. The CAG promoter is a synthetic promoter that combines a modified chicken beta-actin promoter containing the splice acceptor from the rabbit beta-globin gene plus the cytomegalovirus (CMV) IE enhancer ^4^.

To our knowledge, no systematic characterisation of promoter elements has been performed in the DT40 model system. This is a potential limitation to performing successful analysis of gene function and the exploitation of DT40 cells for expression of exogenous proteins: appropriate choice of a promoter element and the resulting protein expression level is of significant importance when developing a suitable model tailored to meet the needs of a particular research project. Thus, to identify suitable expression systems for the analysis of phenotypes upon protein expression, and potentially to aid exogenous protein production from DT40 cells, we compared the widely used CMV promoter with the CAG (CMV early enhancer and chicken beta actin) and CBA (chicken beta actin promoter including the non-coding exon 1 and intron 1 of the beta actin transcript) elements for their respective ability to drive expression of a *TOPBP1* transgene. The choice of these particular promoters was based on previous reports describing them as being among the stronger constitutive promoters available ^5^. Additionally, the CBA promoter has been successfully used for the overexpression of an *ATR* transgene integrated within the *OVA* locus and the subsequent disruption of the endogenous *ATR* locus of DT40 cells ^6^.

Analysis of CMV-, CBA- and CAG-dependent TopBP1 protein levels in an isogenic DT40 background identified CAG as the most efficient promoter element and confirmed the *OVA* locus as an appropriate integration site. The constructs and data generated from our work establishes a stably integrated overexpression system (SIOS) useful for the overexpression of the *TOPBP1* transgene that can be adapted to express any protein of interest. We show that expression from the SIOS is stable over several generations, and thus provides a useful experimental system that can be adapted for a variety of purposes in the DT40 field, including protein production for biochemical studies and the generation of knock-out cell lines expressing a mutated gene of interest. We also show that our SIOS constructs can be excised from the genome by tamoxifen-induced Cre recombination ^7^.

## Methods

### DT40 Cell Culture

DT40 cells (obtained from the Shunichi Takeda’s laboratory, Tokyo, Japan) were cultured in RPMI1640 medium supplemented with 10% heat inactivated fetal calf serum (FCS), 1% chicken serum (Gibco BRL, Grand Island, HY, USA), 10 ^−5^ M beta-mercaptoethanol, 50U/ml penicillin and 50μg/ml streptomycin at 39.5° and 5% CO _2_. Cells were passaged by diluting 1:10–1:20 into fresh media every 1–2 days to maintain the cells in exponential growth phase.

### Generation of targeting constructs

To assemble the ovaCMV construct, a linear DNA fragment containing the CMV promoter, a polyA tail, the SV40 promoter, neomycin gene and a second polyA tail was synthesized (Genscript) and cloned into pUC57 using the *Eco*RV cloning site. To ensure that the expression system being developed would be functional, the nucleotide sequences of the aforementioned genetic elements were copied from widely used mammalian expression vectors: the CMV promoter and the polyA tail sequences were copied from the pCDNA3.1 (V790–20, Addgene), the SV40-neomycin-polyA fragment was copied from pCI (E1841, Addgene). Unique cloning sites were introduced to allow for subcloning of the ovalbumin left arm (ovaLA) as a *Nhe*I/ *Sal*I fragment, the ovalbumin right arm (ovaRA) as a *Asc*I/ *Xho*I and the *TOPBP1* cDNA as a *Not*I/ *Xma*I fragment. The pairs of primers used for these three cloning steps were P1/P2, P3/P4 and P5/P6, respectively (see Table 1). Each cloning step was monitored by diagnostic digestion and, following the final cloning step, Sanger sequencing was performed.

**Table 1.** Primers used in this study.

Primer	Sequence (5’-3’)
P1	GCGTCGAGATGCTAGCGAGGCTCACCTGGACTTCATATCCTTTTGG
P2	ACGAAGTTATGTCGACGGATGGGAGAGAAGACTGGGAAATATTG
P3	TAAGCAGGCGCGCCTAGTTCTGGGACAGTTTGCTACCC
P4	TGCTTACTCGAGGGTACCTCTTTCTATGCATTTTATCCCTACCA
P5	CAAGCTGGCGCGGCCGCATGAAAGGCAGCAAGGAGGTGTTCTT
P6	TTTAAACTGACCCGGGTCAGTGCATTCTGGATCGCTTGA
P7	TCGAATTCAAAGGAGGTACCCACCATGGGGCGCATGAAAGGCAGCAAGGAGGTGTT
P8	GAGGTAGATATCGCGGTACCTCAATGCATCCGGCTCCTTTTTACTCTGCTCATTTCTCCCGGTGCTTTTC
P9	GGGCTATCGAAACTTAATTAAAGAACCAGCTGTGGAATGTGTGTC
P10	CTGACTTGACTGGTTAATTAAGGTACCTCTTTCTATGCATTTTATCCCTACCA
P11	ACCTTTTTGGCAGCGATCGGAGCTCACCTGGACTTCATATCCTTTTGG
P12	ATGTCGGGAGCCGCGATCGATAACTTCGTATATAATACCATATACGAAGTTATGTCGAC

To assemble the ovaCBA and ovaCAG constructs, the *TOPBP1 cDNA* was sub-cloned into two commercially available expression vectors, pSF-CBA (OG262) and pSF-CAG (OG505) at the *Kpn*I site. The SV40-neomycin-pA-loxP-ovaRA fragment from ovaCMV was introduced at the *Pac*I site and the ovaLA-loxP fragment was introduced at the *Pvu*I site. The final constructs were verified by Sanger sequencing. The primers used were P7/P8 (see Table 1).

### Stable targeted transfection of DT40 cells

20μg of the targeting vector were linearized with the restriction enzyme and purified by ethanol precipitation. OvaCMV was linearized with *Xho*I. ovaCBA, ovaCAG and eCAG were linearised with *Apa*LI. Linearised DNA was used for electroporation into a *TOPBP1 ^flox/flox/+^* cell line in which the entire coding region of for TopBP1 had been removed from two of the three alleles present in DT40 cells. 5–10×10 ^6^ DT40 cells were centrifuged at 1500 rpm for 5 minutes at room temperature and the cell pellet was carefully resuspended in 0.5ml chilled PBS. Linearized plasmid was mixed with the cell suspension, transferred to an electroporation cuvette (Biorad, #1652088) and incubated on ice for 10 minutes. Cell electroporation was performed in the Gene Pulser Xcell total system at 550V and 25μF. The cuvette was subsequently incubated on ice for 10 minutes. The transfected cell suspension was next transferred to a flask containing 20ml fresh pre-warmed RPMi media and incubated at 37°C overnight. Selection was performed with 2mg/ml G418. Cells were plated into 96-well plates to isolate single clones. Cells that successfully formed colonies following selection were subjected to Western blot analysis with an anti-FLAG antibody to visualize ectopic TopBP1-3XFLAG.

### SDS-PAGE and immunoblotting

Whole cell protein extracts were separated by sodium dodecyl sulphatepolyacrylamide gel electrophoresis (SDS-PAGE). Gels were run in a BIORAD Mini-POTEAN TetraCell or a C.B.S Double or Triple-wide electrophoresis system in 1x SDS running buffer (0.025M Tris Base, 0.25M Glycine, 0.1% SDS) at 80 volts constant through the stacking gel and 100–120 volts through the separating gel. Proteins were transferred to a Nitrocellulose blotting membrane (#10600001, GE Healthcare) by wet blotting in a transfer cassette (#EBU-302C.B.S Scientific). The transfer was performed in 1x transfer buffer (25mM Tris, 190mM glycine, 10% methanol). The membranes were probed using the appropriate antibodies in 3% milk/PBST for 1h at room temperature. Antibodies used were: mouse monoclonal anti-FLAG (Sigma-Aldrich Cat# F1804 Lot# RRID: AB_262044) used at 1:3000, mouse monoclonal anti-alpha tubulin (Sigma-Aldrich Cat# T5168 Lot# RRID: AB_477579) used at 1:5000, and polyclonal rabbit antiserum specific for avian TopBP1. This was generated using a synthetic peptide corresponding to the C-terminus of chicken TopBP1 conjugated to keyhole limpet haemocyanin. A recombinant GST-tagged peptide corresponding to the last 200 amino acids of chicken TopBP1 was used to purify the anti-TopBP1 antibody from the immune serum. 5ml *E. coli* harbouring the expression plasmid was grown at 37°C to saturation, added to 1 litre LB-Amp and grown to an OD _595_ of 0.6. IPTG was added to final concentration of 0.5mM and cells grown overnight at 20°C. Cells were pelleted by centrifugation, and resuspended in 35ml PBS plus 1 protease inhibitor tablet (Roche) and 1 mM final concentration of AEBSF. The cell mixture was sonicated on ice and cleared by centrifugation. Cell lysate was added to Glutathione Sepharose beads (1ml washed with water to remove the ethanol and equilibrated in PBS) and incubated for 1h at 4°C with gentle agitation. The beads were washed three times with 30ml of PBS and twice with 10ml of 0.2M sodium borate pH9. Dimethylpimelimidate (DMP) was added to 20mM and gently mixed for 1h at room temperature to cross-link. To terminate cross linking the beads were harvested, resuspend in 10 ml of 0.2M Tris pH 8 and incubated for two hours at room temperature, rolling. The beads were harvested and washed once with 5ml of 0.1M glycine-HCl pH2.5 and twice with 10ml PBS.

For antibody purification, 9ml of serum were mixed with 1ml of 10x PBS and incubated with 1ml GST-linked resin at 4°C for 4h with rolling. The mixture was applied to a column and washed once with 10ml PBS, once with 10ml TrisHCl pH7.5/250mM NaCl and once with 10ml TrisHCl pH7.5/750mM NaCl. Antibody was eluted with triethylamine pH11.5 and equilibrated with TrisHCl pH5 to adjust the pH to 7. The eluted serum was re-applied to the column (after the column was washed extensively with PBS) and the above process repeated three times. The purified anti-TopBP1 antibody was used at 1:1000.

### Immunoblot quantification

Western blots were quantified by densitometry and ImageJ software (version 1.51j8). Background-subtracted densities of the band (protein) of interest (BI) as well as the normalising control (NC) were determined. Subsequently all the NC values were divided by the NC value of the highest density such that the latter is equal to 1. This gave rise to the relative NC values (rNC). Finally, each BI value was divided by the rNC value of the respective lane, giving rise to the normalised BI values (nBI).

### Southern blotting

Genomic DNA was extracted using a standard ethanol extraction. DNA concentration was measured using a Nanodrop and the appropriate amount of DNA digested in a final volume of 200μl at 37°C. Digested DNA was isopropanol precipitated and resuspended in 20μl of dH _2_O and loading buffer. The samples were run on agarose gels (concentration dependent on fragment size) in 1× TBE at 50V. The gel was subsequently incubated for 20 minutes in depurinating solution (0.25M HCl) while rocking, washed in denaturing solution (1.5M NaCl and 0.5M NaOH) for 30 minutes rocking and washed in neutralizing solution (1M Tris and 1.5M NaCl). The gel was then transferred to a membrane (GeneScreen #NEF983001PK, Perkin Elmer) employing 10× SSC buffer (1.5M NaCl, 0.15M sodium citrate pH 7) and capillary force over night, washed in 2× SSC buffer for 5 minutes on a shaker, air dried on a piece of filter paper and then the DNA was cross-linked to the membrane using 1200J/m ^2^ UV light.

A specific probe for Southern blot analysis was generated by PCR amplification of a ≈500bp fragment from the locus of interest and gel extraction. For hybridisation the membrane was washed in dH2O for 5 minutes. Next 80ml of preheated 65°C hybridising solution I (6× SSC, 1x Denhardt [100x: 2% Ficoll 400, 300mM NaCl, 2% polyvinylpyrrolidone, 2% BSA], 1% sarcosyl, 0.1% BSA) was added to the hydrated membrane and incubated for hour at 65°C. To label the probe 1μl of 50ng/μl DNA was added to 44μl dH _2_O, boiled for 5 minutes and quenched on ice. Rediprime II Random Prime system (#RPN1633, GE Healthcare) and 5μl of 32P-αdCTP were added to the DNA and the mixture was incubated at 37°C for 15 minutes. The labelled probe was then spun in a pre-spun G50 column at 3000rpm for 1 minute and incubated at 100°C for 5 minutes and quenched on ice. The probe was added to 20ml preheated 65°C hybridising solution II (6× SSC, 1x Denhardt, 1% sarcosyl, 200μl 10mg/ml salmon sperm DNA). Hybridising solution I was replaced with hybridising solution II and incubated at 65°C over night. The following day the membrane was washed with 500ml preheated wash buffer I (2× SSC, 1% SDS) at 65°C shaking for 15min. The membrane was subsequently washed twice, with 500ml of buffer II (0.1× SSC, 0.1% SDS) at 42°C, shaking for 15 minutes, then air dried on tissue, wrapped in cling film and placed in a phosphoimager cassette overnight. The screen was scanned to obtain the Southern blotting results.

### Flow Cytometry

500μl of mid logarithmically growing cells were washed with PBS and fixed in 1ml ice-cold 70% ethanol/PBS while vortexing to minimise the formation of clumps and ensure uniform cell fixation. Fixed cells were stored at 4°C overnight or at -20°C for between two hours to several weeks before further analysis. Fixed cells were centrifuged at 1500rpm for 5min and the cell pellet washed twice with 3% BSA/PBS. Cells were resuspended in 500μl of 3% BSA/PBS containing 250 μg/ml Ribonuclease A (RNase) and 10μg/ml propidium iodide (PI) (Sigma #81845). Samples were left for one hour at room temperature in the dark or at 4°C overnight before analysis on a BD FACSCanto machine (BD) using the FL-A setting. Analysis was performed on BD Accuri C6 Software (version 1.0.264.21).

### Growth curves

1 × 10 ^5^ cells were seeded in pre warmed growth media and counted after 24h. The culture was split 1/10 to the same volume, left to grow for another 24h and counted again. The procedure was repeated for the specified number of days. Raw data were processed in Microsoft Excel (2016).

### Excision of floxed-DNA sequences by induction of Mer-Cre-Mer

10 ^5^ cells transfected with floxed vectors were cultured in 1ml of growth medium containing 2μM 4-hydroxytamoxifen (4-HT) for 24h and subcloned with limiting dilution using final concentrations of 30, 100, 300 and 1000 cells per 96-well plate. 6–8 days after subcloning, single clones can be observed as single colonies on the bottom of the plate. To expand single clones, 10μl of stable transfectants were transferred into 1ml of growth medium.

## Results

### Relative promoter activities of *TOPBP1* transgenic constructs stably integrated at *OVA*

To avoid the high variation in transgene expression resulting from uncontrolled copy number and chromosomal position effects when using non-targeted integration, we targeted the CAG, CBA and CMV expression systems into the *OVA* locus in a *TOPBP1 ^flox/flox/+^* cell line. This cell line was created by sequentially transfecting two separate but related constructs (Puromycin resistant and histidinol resistant) containing 3’ (2Kb) and 5’ (4kb) homology arms and validating the correct integration events by Southern blot analysis. The resistance markers were subsequently removed by inducing Cre recombination after random integration of sequences encoding a tamoxifen-regulated chimeric MerCreMer enzyme ^7^. The resulting *TOPBP1 ^flox/flox/+^* cell line has two of the three copies of the *TOPBP1* gene deleted (Δ introns 2 –27, designated “ *flox*”) and also expresses a tamoxifen regulated chimeric MerCreMer enzyme. The MerCreMer version of the Cre recombinase protein is inactive due to its retention in the endoplasmic reticulum in the absence of oestrogen derivatives. Creating multiple isogenic clones in this cell line that are constitutively overexpressing the *TOPBP1* transgene from three different promoters allowed a direct comparison of constructs within the same genomic context to provide a systematic quantitative assessment of the strengths of the promoters.

**Figure 1.**
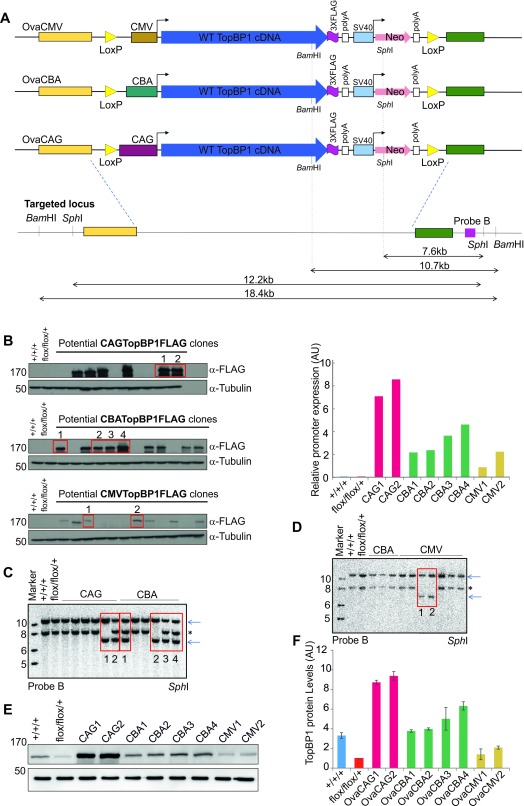
Relative promoter activities of *TOPBP1* transgenic constructs stably integrated at *OVA*. **A**) Schematic representation of *TOPBP1* expression constructs under the control of CMV, CBA and CAG promoters and of the *OVA* locus. **B**) Immunoblot analysis using α-FLAG to identify TopBP1-3xFLAG in cell lysates from transfected cells. Cell lysates of non manipulated DT40 and parental ( *TOPBP1* ^*flox/flox/*+^) cells were used as a negative controls. Beta-tubulin was used as a loading control. Correctly integrated clones (see **C** and **D**) are indicated by the red boxes and numbered. Right: quantification of FLAG signal for numbered clones. Quantification was performed as described in the Materials and Methods. TopBP1-Flag expression for +/+/+ and *TOPBP1* ^*flox/flox/*+^ untransfected controls was set equal to 0. **C**, **D**) Clones showing expression of the *TOPBP1* transgene in **B** were subjected to Southern blot analysis following *Sph*I digestion of genomic DNA. The diagnostic bands corresponding to the wild-type allele (12.2kb) and the successfully targeted allele (7.6kb) are indicated by the red boxes. The numbers below the boxes correspond to the numbered clones in **B**. The asterisk corresponds to one of the three *OVA* alleles containing a polymorphism that generates an *Sph*I restriction site. The 3’ probe used is shown in **A** (purple bar). **E**) Representative western blot analysis of cell lysates from cells expressing the *TOPBP1* transgene. Immunolotting with an α-TopBP1 antibody allows the visualisation of total TopBP1 protein levels, facilitating comparison to the endogenous promoter. Beta-tubulin serves as the loading control. **F**) Quantification of three equivalents to the blot shown in **E** to show the increase of TopBP1 protein levels over the levels produced from the endogenous promoter. Quantification was performed as explained in the Materials and Methods. The nBI value for *TOPBP1* ^*flox/flox/*+^ was set equal to 1 and all other nBI values calculated relative to that.

The three constructs assembled for the integration of the three expression cassettes at the *OVA* locus are shown in Figure 1A. The targeting experiments and subsequent analysis were performed in parallel to eliminate variations due to the experimental conditions. As shown in Figure 1B, most of the G418 resistant clones obtained were positive for the expression of the FLAG-tagged TopBP1. To distinguish the clones that have targeted the construct correctly at the *OVA* locus, Southern blot analysis of *Sph*I-digested genomic DNA was performed. Hybridisation with an appropriate probe (probe B, Figure 1A) revealed those TopBP1-FLAG expressing clones that were integrated at the *OVA* locus. This is evident from the 7.6kb band that corresponds to a successfully targeted *OVA* allele ( Figure 1C,D, positives in red boxes). FLAG-tagged protein levels were quantified in the correctly targeted clones ( Figure 1B, right)

Two clones of each of *TOPBP1 ^flox/flox/+^ OVA ^CMVtopbp1/+/+^, TOPBP1 ^flox/flox/+^ OVA ^CBAtopbp1/+/+^* and *TOPBP1 ^flox/flox/+^OVA ^CAGtopbp1/+/+^* were subjected to PCR amplification of ~0.7kb partially overlapping fragments which were subjected to Sanger sequencing. This ensured that no mutations had been incorporated across the length of the transgene during integration. Having the transgene fused to an epitope-tag allowed the characterization of the relative efficiencies of the three distinct promoters among the three isogenic counterparts. As depicted in Figure 1B, right, the CAG promoter displayed the highest strength as judged by FLAG-Tagged TopBP1 protein levels. The average relative expression of the two CAG clones was 7.5AU (arbitrary units), of the four CBA clones: 2.8AU and of the two CMV clones: 1.3AU. Hence, the CAG promoter element showed more than 2.6-fold higher expression of the transgene than CBA and 5.8-fold than CMV.

Probing with an α-TopBP1 antibody ( Figure 1E) enabled a qualitative assessment of the three systems based on the fold-increase of the total protein levels over the amount of endogenous TopBP1 in the parental cell line ( Figure 1F). When compared to exogenous TopBP1, the CMV construct contributes less than 1-fold of the total pool of TopBP1 protein inside the cells, whereas CBA and CAG contribute to approximately 2- and 5- fold more than the endogenous allele ( Figure 1 E,F). Thus, a single copy of the CBA promoter increases the TopBP1 levels to approximately the levels normally found in wild-type cells (where there are three copies of the endogenous gene) while the CAG promoter provides approximately three times the amount of TopBP1 than that found in wild-type cells. This data confirms the CAG promoter as the most efficient among those tested in driving expression of an ectopic *TOPBP1* in DT40 cells.

### Comparison of CAG-driven TopBP1 expressed from the *OVA* locus with a euchromatic locus

*OVA* is a silent gene in the chicken B cells ^8^, which may affect the expression levels due to the local chromatin environment. To examine if this was the case we compared TopBP1 protein production from the CAG promoter integrated at the *OVA* locus to the levels of TopBP1 when expressed from the same promoter construct integrated at a transcriptionally active locus. To achieve this, the *TOPBP1* transgene construct under the control of the CAG promoter was recreated with different homology arms. We chose to target a region downstream of the endogenous *TOPBP1* locus on chromosome II (42,781,752–42,789,165), a region between the 3’ end of the *TOPBP1* transcriptional unit and the *CDV3* protein-coding gene.

**Figure 2.**
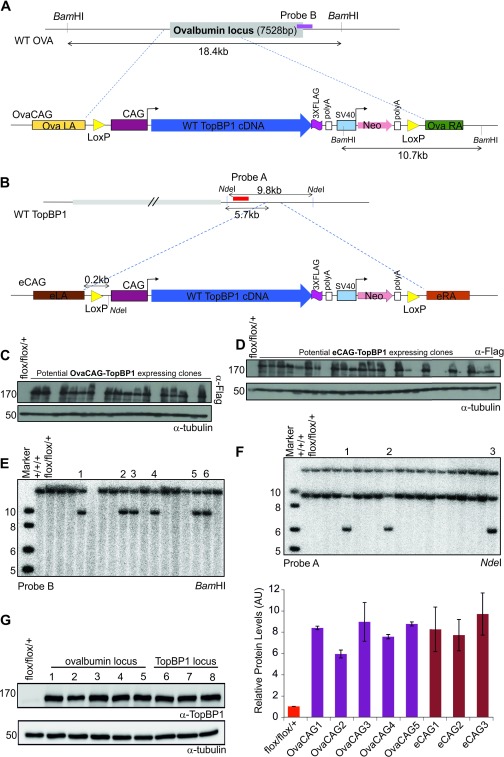
Relative promoter strength of CAG integrated at the *OVA* locus versus a euchromatic locus. **A**) Schematic representation of the *OVA* locus and the targeting construct used to stably integrate the CAG- *TOPBP1* transgene at the *OVA* locus. **B**) Schematic representation of the *TOPBP1* locus and the targeting construct used to stably integrate the CAG- *TOPBP1* transgene adjacent to the *TOPBP1* gene. **C**) Immunoblot analysis using α-FLAG to identify TopBP1-3xFLAG in cell lysates from cells transfected with the ovaCAG construct shown in **A**. Cell lysates of non manipulated DT40 and parental ( *TOPBP1* ^*flox/flox/*+^) cells were used as a negative controls. Beta-tubulin was used as a loading control. **D**) Immunoblot analysis using α-FLAG to identify TopBP1-3xFLAG in cell lysates from cells transfected with the eCAG construct shown in **B**. Cell lysates of non manipulated DT40 and parental ( *TOPBP1 ^flox/flox/+^*) cells were used as a negative controls. Beta-tubulin was used as a loading control. **E**) TopBP1-3xFLAG positive clones from **C** subjected to Southern blot analysis to check from integration at the *OVA* locus. The diagnostic bands following *Bam*HI digestion correspond to the wild-type allele (18.4kb) and the successfully targeted allele (10.7kb). The probe used is shown in **A** (purple bar). **F**). TopBP1-3xFLAG positive clones from **D** subjected to equivalent Southern blot analysis to check for integration adjacent to the *TOPBP1* gene. The diagnostic bands following *Nde*I digestion correspond to the wild-type allele (9.8kb) and the successfully targeted allele (6kb). The probe used is shown in **B** (red bar). **G**) Representative immunoblot analysis using α-TopBP1 of cell lysates from cells where ovaCAG (1–5) or eCAG (1–3) constructs are stably integrated at the respective loci (see **E**, **F**). The increase of TopBP1 protein levels over the levels produced from the endogenous promoter are quantified (right panel). Quantification was performed as explained in the Materials and Methods.

The euchromatin CAG (eCAG) construct was assembled in a similar manner as the original ovaCAG (see Materials and Methods) such that the only difference is the sequence of the left and right homology arms ( Figure 2A,B). The final construct was verified by diagnostic enzyme digestion and Sanger sequencing. The construct was linearized and transfected into the *TOPBP1 ^flox/flox/+^* cell line. To ensure a direct comparison of CAG promoter element when integrated at the *OVA* locus and the locus immediately downstream of the endogenous *TOPBP1* locus, the ovaCAG targeting vector was transfected in parallel to the eCAG targeting vector ( Figure 2B). G418 resistant clones for both transfections were isolated following selection and checked for expression of the TopPB1-FLAG protein by Western blot analysis and α-FLAG antibody. The majority of clones were positive for TopBP1-3xFLAG expression ( Figure 2C, D). To test which of these had the transgenes integrated at the loci of interest, Southern blot analysis was performed. Six out of 16 TopBP1-FLAG positive clones had specifically targeted the *OVA* locus ( Figure 2E) and three out of 17 had successfully targeted the locus proximal to the endogenous *TOPBP1* ( Figure 2F).

The expected sizes for the successfully targeted *OVA* locus have been described above. For the *TOPBP1* proximal locus, *Nde*I digestion of genomic DNA and hybridization with an appropriate probe ( Figure 2B, probe A) generates a 9.8kb band for the *TOPBP1* proximal locus and a 6kb band for clones that have successfully integrated the eCAG construct ( Figure 2F). The additional band above 10kb is likely to be a polymorphism within one of the three alleles on chromosome II as it was present in all the cell lines, including untransfected controls ( Figure 2F).

To estimate the potential differences in expression between the two loci, total protein was prepared from five ovaCAG and the three eCAG integrants, subject to Western blotting and probed with α-TopBP1 ( Figure 2G). The data indicate that there is no major difference when comparing ovaCAG and eCAG, suggesting that the *OVA* locus is an appropriate site for integrating exogenous genes for expression. The main advantage of the *OVA* locus is the high integration rate.

### Stability of SIOS over time

**Figure 3.**
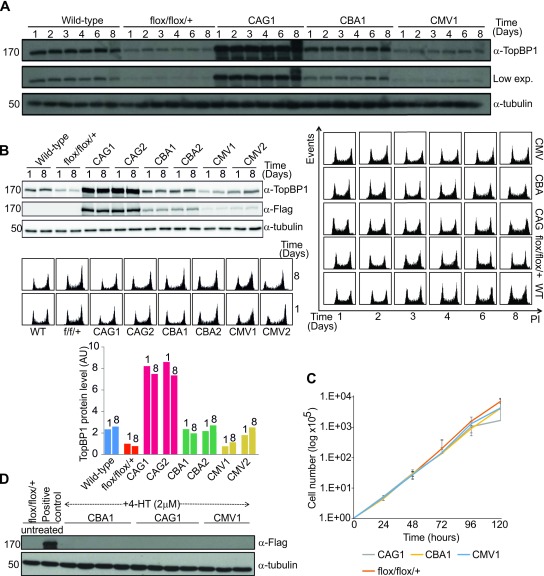
Stability of the SIOS system. **A**) The indicated cell lines (as designated in Figure 1) were cultured for a period of eight days and samples were processed for immunoblot analysis with α-TopBP1 at the specified times. Cell lysates of non-manipulated and parental ( *TOPBP1* ^*flox/flox/*+^) cells were used as controls. Beta-tubulin was used as a loading control. Bottom-right: flow cytometry analysis of samples fixed and stained with propidium iodide at the indicated times. **B**) A repeat of **A** with two independent clones for each promoter. Samples for the 1 and 8 day time points were immunoblotted for both TopBP1 and FLAG. Beta-tubulin was used as a loading control. Middle: flow cytometry analysis of samples fixed and stained with propidium iodide at the indicated times. Bottom: quantification of the TopBP1 protein levels (AU: arbitrary units). **C**) Growth curves for the indicated cell cultures. **D**) The indicated TopBP1 overexpressing cell lines were tested for the ability to flox the *TOPBP1* transgene following treatment with 4-HT (2μM) for 24h. Treated cells were serially diluted and single clones subsequently expanded and analysed by immunoblotting with an α-FLAG antibody. Lysates of parental ( *TOPBP1* ^*flox/flox/*+^) cells not containing the transgene serve as a negative control and lysates from *TOPBP1* ^*flox/flox/*+^ *OVA* ^*CAG-TOPBP1/*+/+^ cells serve as a positive control. The levels of beta-tubulin were used as a loading control.

While it is good practice to freeze cell lines down soon after their production and to perform analyses on freshly recovered samples, we wanted to establish if growing cells for multiple generations could result in suppression of the promoter, for example by methylation. The CMV, CAG and CBA overexpression systems integrated at the *OVA* locus were thus characterised in terms of their stability. The TopBP1 expression levels of representative cell lines for each of the CMV, CAG and CBA constructs were followed for eight days in culture. This continued culturing did not affect the promoters’ activity, as judged from Western blot analysis and immunoblotting against TopBP1 ( Figure 3A). We followed the cell cycle profiles by flow cytometry to establish if the overexpression of TopBP1 was influencing the progression of the cell cycle. No changes were observed over the course of the eight days ( Figure 3B). In addition, when compared to the parental strain, no growth defect was associated with overexpression of the *TOPBP1* cDNA when cell numbers were followed over a 120 hour period ( Figure 3C). Finally, induction of Cre recombinase by 4-HT treatment for 24h and subsequent serial dilution to isolate single clones confirmed that the ectopic transgenes could successfully be floxed ( Figure 3D).

## Discussion

In this report, we have characterised the relative strengths of three promoter elements, CMV (cytomegalovirus), CAG (CMV early enhancer and chicken beta actin) and CBA (chicken beta actin) in DT40 cells. Our data adds to a relatively sparse literature comparing promoter strengths in DT40 cells ^9, 10^. We demonstrate that CMV is a relatively weak promoter in DT40, producing approximately the same quantity of an exogenous TopBP1 reporter protein as is produced from a single copy of the native TopBP1 gene. CBA shows several fold higher activity while CAG showed the highest level of activity in our assay, resulting in approximately 7–8 fold more protein than a single endogenous *TOPBP1* gene. We did observe variation between individual clones for a specific promoter construct, but this did not exceed two fold. Importantly, the level of protein produced from each of the three promoters was not altered following growth for 8 days. This data suggests that integration of these promoters in DT40 does not result in silencing over this timeframe and thus these are suitable tools for protein expression studies.

The *OVA* locus is commonly used for protein expression in DT40 cells because it is transcriptionally silent in this cell line and it shows a high targeting efficiency compared to multiple other loci studied ^8^. However, it was not clear if the silent nature of the locus might affect the efficiency of promoters that are integrated at this locus. Here we have compared the levels of TopBP1 produced from a CAG-driven transgene at the OVA locus (ovaCAG) with the same transgene and promoter integrated at a euchromatic site (eCAG). Although we did observe that individual clones showed modest variation within each of the two loci (not exceeding two fold), we did not see any major difference between the ovaCAG and eCAG clones. From this we conclude that the *OVA* locus is an appropriate choice for integration.

Finally, we report SIOS constructs for integration of a chosen gene into the *OVA* locus under control of one of three promoters with various strengths and demonstrate that our constructs can be excised by induction of Cre recombinase by 4-HT treatment.

## Supplementary material

**Supplementary File S1: Uncropped blots for Figures 1B, 1C, 1D, 1E, 2C, 2D, 2E, 2F, 2G, 3A, 3B and 3D.**
