# Supplementary material for: Characterisation of a stably integrated expression system for exogenous protein expression in DT40
Source: Wellcome Open Res. 2017 Dec 12;2:40. Originally published 2017 Jun 8. [Version 2] doi: 10.12688/wellcomeopenres.11816.2 (PMC5482329; doi:10.12688/wellcomeopenres.11816.2)

1B Tubulin

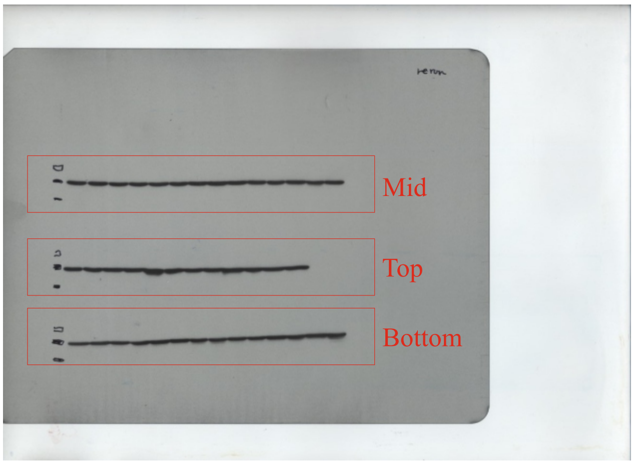

Flag

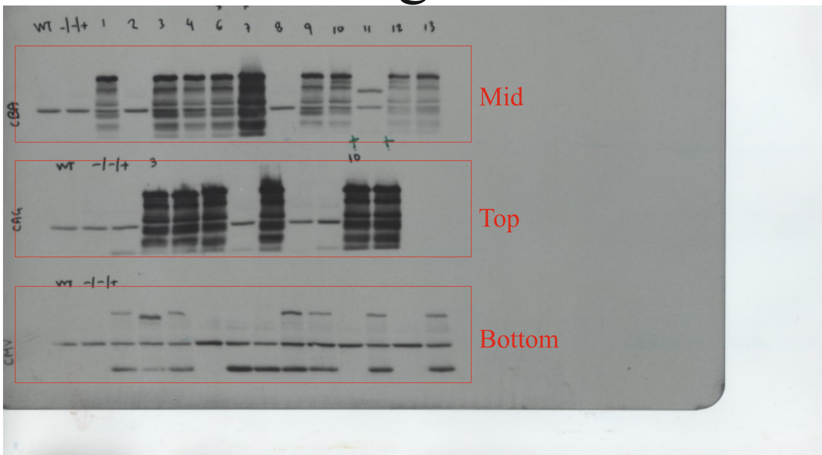

1C

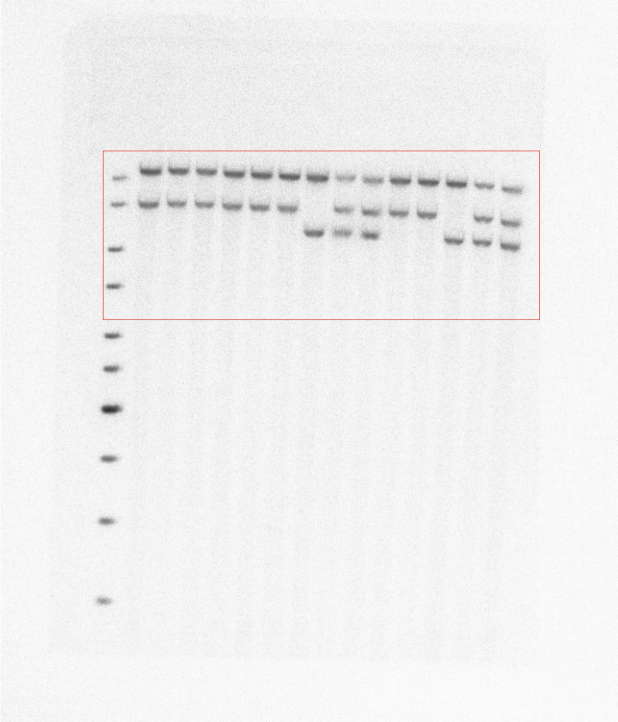

1D

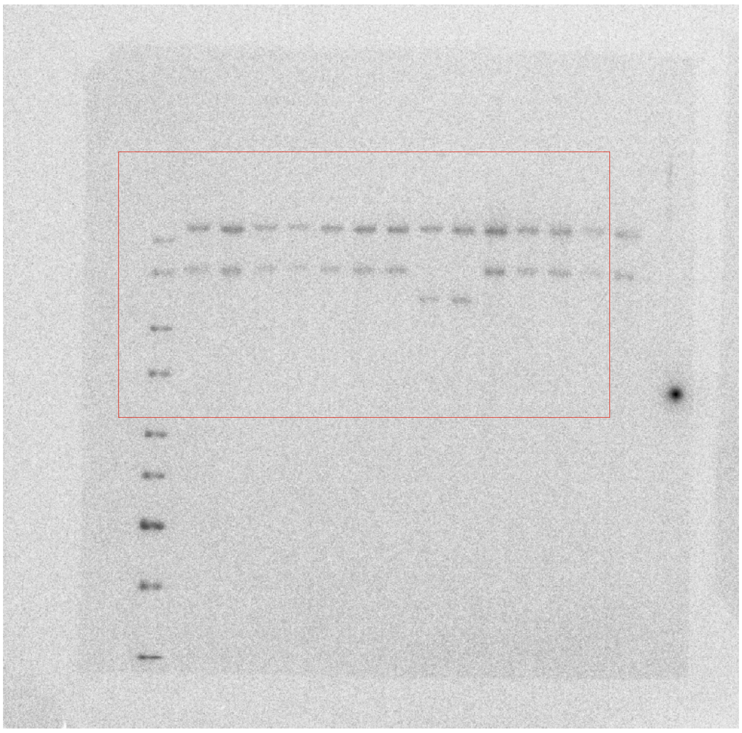

1E TopBP1

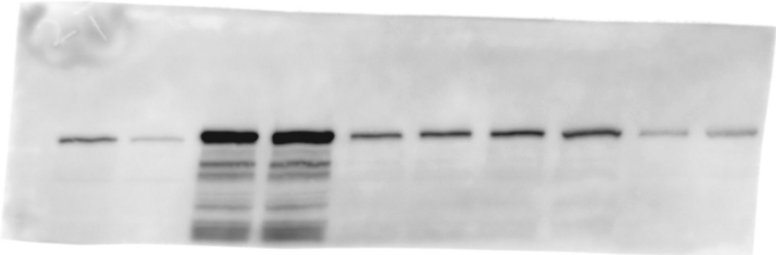

Tubulin

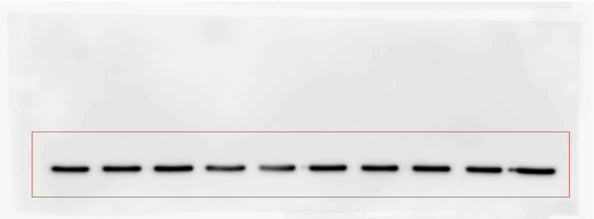

2C,D FLAG

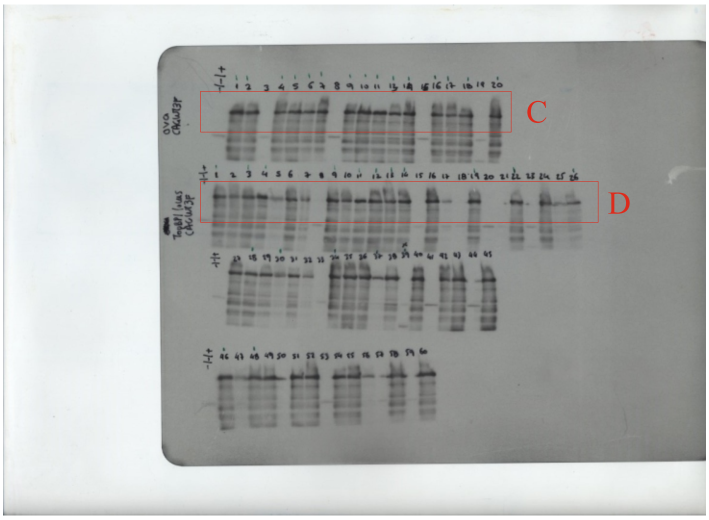

Tubulin

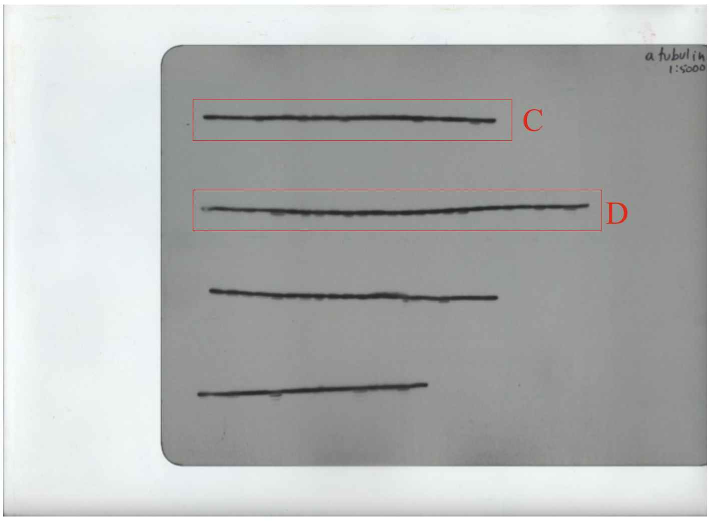

2E

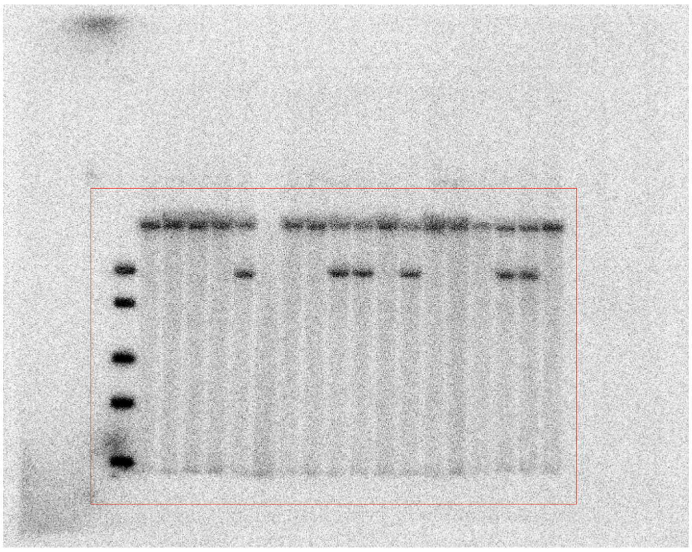

2F

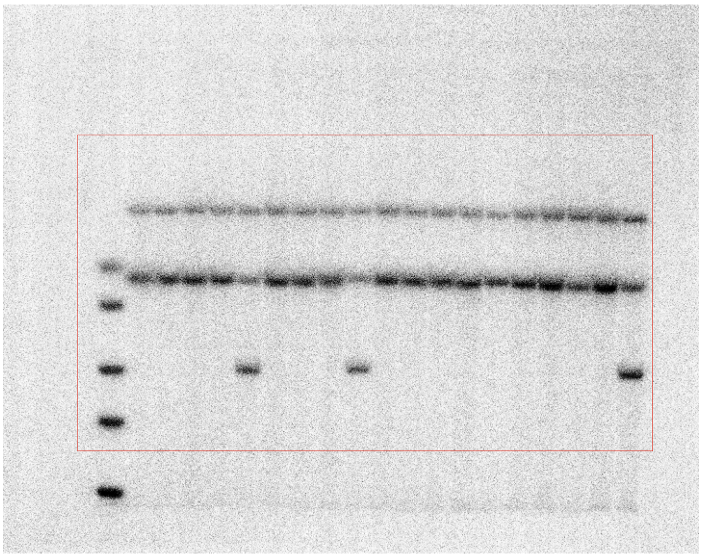

2G TopBP1

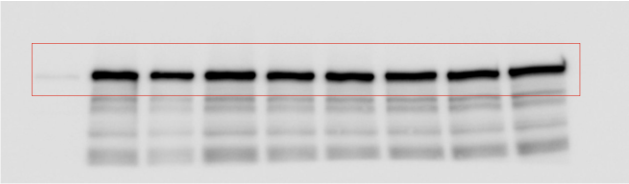

Tubulin

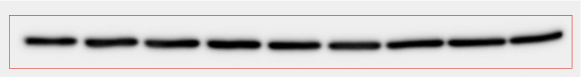

3A

TopBP1

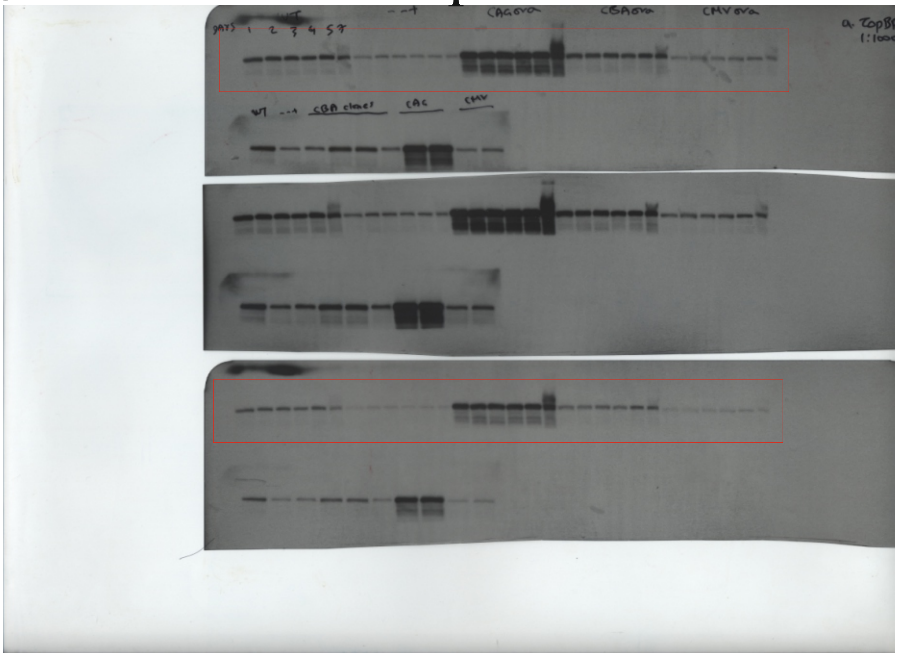

Tubulin

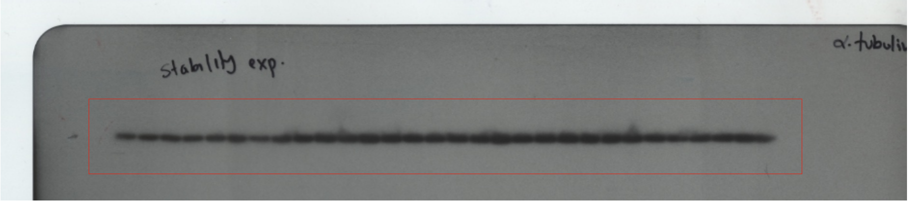

3B

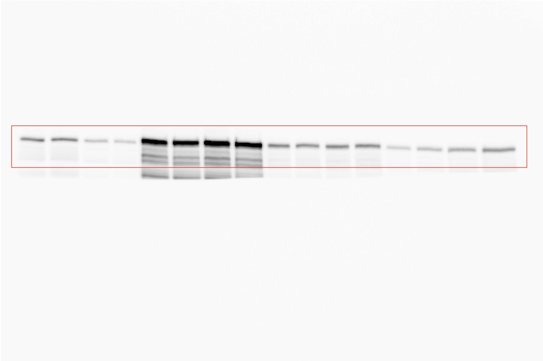

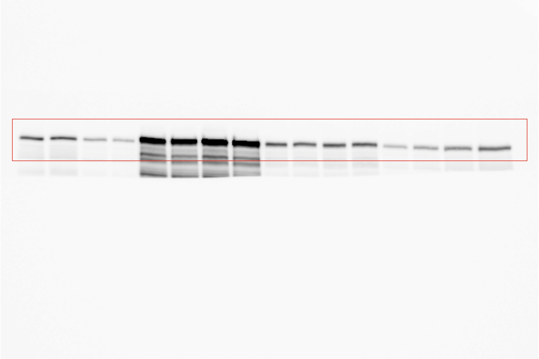

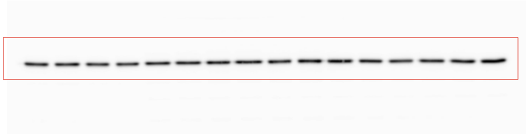

3D

TopBP1

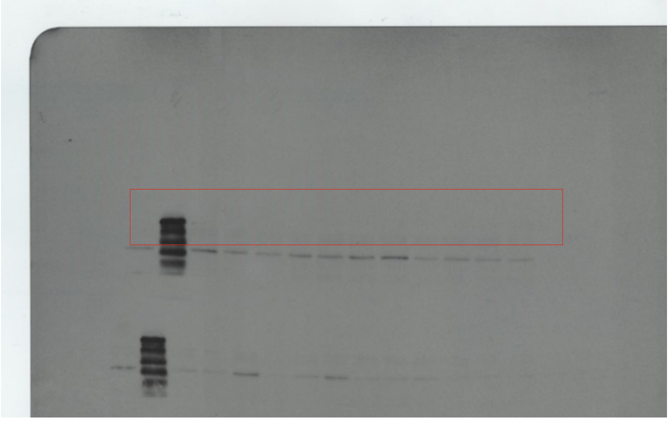

Tubulin

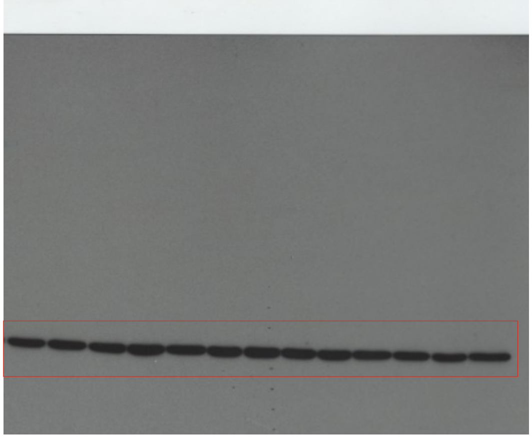

Supplement: Supplementary file 1 [file wellcomeopenres-2-14617-s0000.tgz › d52dca1d-4438-4a14-b484-952368055141.pdf]
