# Supplementary material for: Characterisation of a stably integrated expression system for exogenous protein expression in DT40
Source: Wellcome Open Res. 2017 Dec 12;2:40. Originally published 2017 Jun 8. [Version 2] doi: 10.12688/wellcomeopenres.11816.2 (PMC5482329; doi:10.12688/wellcomeopenres.11816.2)
Supplement: Peer review history

# Peer review: Referee response for version 2

**DOI:** 10.21956/wellcomeopenres.14617.r28976

**Published:** 2017-12-19

**Authors:** Ciaran G. Morrison

**Affiliations:** 1 Centre for Chromosome Biology, School of Natural Sciences, National University of Ireland Galway (NUI Galway), Galway, Ireland

The revised submission from Skouteri and colleagues addresses the principal issues of my initial review. The data are now presented in a more convincing manner and there is more indication of the reproducibility of the experimental findings. The study now presents a useful comparison for promoters to drive transgene expression in the DT40 system.

I have read this submission. I believe that I have an appropriate level of expertise to confirm that it is of an acceptable scientific standard.

# Peer review: Referee response for version 1

**DOI:** 10.21956/wellcomeopenres.12770.r23336

**Published:** 2017-06-20

**Authors:** Julian E. Sale

**Affiliations:** 1 Medical Research Council Laboratory of Molecular Biology, Cambridge, UK

DT40 cells remain a powerful and versatile model for vertebrate somatic cell genetics. Ectopic expression of proteins is an important tool, for example for complementation studies. Random integration of expression constructs is most frequently used, but suffers from variability largely due to the influence of local chromatin environment. There has also been some debate about the best heterologous promoters to use in DT40.

 This methods paper explores the use of three promoters in expression constructs knocked into two distinct loci, one heterochromatic and one euchromatic. The results are pretty clear: the CAG promoter gives the highest levels of expression but, interestingly, performs equally well in the repressive environment of the ovalbumin locus as in the euchromatin near *TOPBP1*.

 This is a very useful study and should help improve consistency in complementation studies in DT40 by showing that the *OVA* locus can be used similarly to *ROSA26* in mice. I have only a few minor suggestions to improve the presentation of the results: 

1. It would be helpful to define the acronyms used for the promoters at the start, preferably in the abstract.
2. The labelling of Figure 1B – G could be improved to make it clear on the Western blots those clones that are expressing from a correctly integrated construct and those that are from random integrants. Use of numbering would help link the Westerns and Southerns. Incidentally, the red boxes in Figure 1 are not mentioned in the legend. Indeed, it might be worth pointing out that the SIOS constructs will still work if not correctly integrated, and that some assessment of correct targeting is needed alongside testing for expression.
3. Some of the font sizes on the Figures need attention e.g. the graph in Figure 2G.
4. ‘experimental’ is redundant in the last sentence of the abstract.

I have read this submission. I believe that I have an appropriate level of expertise to confirm that it is of an acceptable scientific standard.

# Peer review: Referee response for version 1

**DOI:** 10.21956/wellcomeopenres.12770.r23338

**Published:** 2017-06-20

**Authors:** Shunichi Takeda, Mahmoud Abdelghany Gomaa Ibrahim

**Affiliations:** 1 Department of Radiation Genetics, Department of Radiation Genetics, Kyoto University, Kyoto, Japan; 2 Department of Radiation Genetics, Kyoto University, Kyoto, Japan

The article is well written, innovative in terms of providing systemic characterization for promotor elements in DT40 model system and identifying CAG as the most efficient promotor element, however they made constructs only for expressing TopBP1. If they add data showing the efficiency of their system in DT40 cells for exogenous expression of other genes, the value of this paper will be increased.

We have read this submission. We believe that we have an appropriate level of expertise to confirm that it is of an acceptable scientific standard.

# Peer review: Referee response for version 1

**DOI:** 10.21956/wellcomeopenres.12770.r23337

**Published:** 2017-06-16

**Authors:** Ciaran G. Morrison

**Affiliations:** 1 Centre for Chromosome Biology, School of Natural Sciences, National University of Ireland Galway (NUI Galway), Galway, Ireland

**Major comments**

 Skouteri and colleagues test the transgenic expression of TopBP1 from 3 promoters at the chicken *OVA* locus or at an adjacent region in DT40 cells. Effectively, this reads as a limited set of optimisation experiments for a standardised integration site for transgene expression. A more completely cross-factored analysis could be useful but the limited choice of parameters explored here makes it difficult to be convinced even that the CAG promoter is the most efficient for expression at OVA-integrated transgenes in DT40.

1. Reproducibility is not demonstrated convincingly. Quantitation from the immunoblots in Fig 1B and 1C seems to be based on a single experiment, with a markedly limited sample size (2-4 clones, obtained in individual rounds of targeting and examined once, from what is described in the MS.). This is not convincing in terms of general applicability. The fluctuations in CBA2 and CBA4 expression levels between Fig 1C and 1G indicate the need for repeated analyses and larger number of samples. Similar issues apply to Figure 2.
2. The use of tagged TopBP1 as a readout of promoter efficacy in TopBP1(+/+/-) cells is problematic. This is not a reporter gene but one whose expression levels influence cell status. Even though the transgene expression level does not vary much once established, as in Fig. 3 A, these clones have already been clonally selected, possibly for their expression level. This is a difficulty in generalising the findings presented here to other transgenes of interest.  A similar set of experiments with reporter genes would obviate this potential concern.
3. Stability of transgene expression is another issue of great importance in designing SIOS experiments. Some quantitative idea of how well the relative expression levels are retained would be valuable. This is indicated in Figure 3A but for individual clones of each promoter type targeted within the OVA locus, rather than for the entire dataset.  Is this stability clone-dependent or promoter-dependent (i.e., do low-level CAG clones show lower stability than high-level?).

 

**Minor comments**

1. Details of how the 30 kb chicken TopBP1 locus was removed would be useful.
2. Figure panels should be cited in order in the text or positioned appropriately.  Figure 2G is not described in the text.
3. Figure quality should be improved. Variable font sizes and formats, poor labelling of marker positions in immunoblots and unjustified use of 3d bar graphs detract from the overall impression of the manuscript. Furthermore, the immunoblots presented are not of the highest quality; for example, it would be difficult to normalise the FLAG signal with the alpha-tubulin signals shown in Figs. 1B, 1F, 2C, 3A.
4. It is not clear which of the various lines shown in Figs. 1 and/ or 2 are used in Fig. 3D.
5. The Discussion could include mention of the promoter analyses performed in Yang et al. (2006) ^1^, Samejima et al. (2008) ^2^, Vazquez et al. (2003) ^3^.

I have read this submission. I believe that I have an appropriate level of expertise to confirm that it is of an acceptable scientific standard, however I have significant reservations, as outlined above.

## References

[1] YangSYFugmannSDSchatzDG: Control of gene conversion and somatic hypermutation by immunoglobulin promoter and enhancer sequences. *J Exp Med*.2006;203(13) : 10.1084/jem.20061835 2919-28 10.1084/jem.20061835 17178919PMC2118177

[2] SamejimaKOgawaHCookeCAHudsonDFHudsonDMacisaacFRibeiroSAVagnarelliPCardinaleSKerrALaiFRuchaudSYueZEarnshawWC: A promoter-hijack strategy for conditional shutdown of multiply spliced essential cell cycle genes. *Proc Natl Acad Sci U S A*.2008;105(7) : 10.1073/pnas.0712083105 2457-62 10.1073/pnas.0712083105 18263736PMC2268158

[3] VazquezGWedelBJTrebakMSt John BirdGPutneyJW: Expression level of the canonical transient receptor potential 3 (TRPC3) channel determines its mechanism of activation. *J Biol Chem*.2003;278(24) : 10.1074/jbc.M302162200 21649-54 10.1074/jbc.M302162200 12686562
